# White Matter Connectivity of the Thalamus Delineates the Functional Architecture of Competing Thalamocortical Systems

**DOI:** 10.1093/cercor/bhv063

**Published:** 2015-04-21

**Authors:** Jonathan O'Muircheartaigh, Simon S. Keller, Gareth J. Barker, Mark P. Richardson

**Affiliations:** 1 Department of Neuroimaging, Institute of Psychiatry, Psychology and Neuroscience, King's College London, London WC2R 2LS, UK; 2 Department of Molecular and Clinical Pharmacology, Institute of Translational Medicine, University of Liverpool, Merseyside L69 3BX, UK; 3 Department of Radiology, Walton Centre National Health Service Foundation Trust, Liverpool, UK; 4 Department of Clinical Neuroscience, Institute of Psychiatry, Psychology and Neuroscience, King's College London, London WC2R 2LS, UK

**Keywords:** functional connectivity, functional networks, resting-state fMRI, thalamus, tractography

## Abstract

There is an increasing awareness of the involvement of thalamic connectivity on higher level cortical functioning in the human brain. This is reflected by the influence of thalamic stimulation on cortical activity and behavior as well as apparently cortical lesion syndromes occurring as a function of small thalamic insults. Here, we attempt to noninvasively test the correspondence of structural and functional connectivity of the human thalamus using diffusion-weighted and resting-state functional MRI. Using a large sample of 102 adults, we apply tensor independent component analysis to diffusion MRI tractography data to blindly parcellate bilateral thalamus according to diffusion tractography-defined structural connectivity. Using resting-state functional MRI collected in the same subjects, we show that the resulting structurally defined thalamic regions map to spatially distinct, and anatomically predictable, whole-brain functional *networks* in the same subjects. Although there was significant variability in the functional connectivity patterns, the resulting 51 structural and functional patterns could broadly be reduced to a subset of 7 similar core network types. These networks were distinct from typical cortical resting-state networks. Importantly, these networks were distributed across the brain and, in a subset, map extremely well to known thalamocortico-basal-ganglial loops.

## Introduction

The rapid advances of systems neuroscience and growing interest in structure and dynamics of large-scale brain networks adds extra impetus to the need to understand the basic building-blocks of thalamocortical connectivity. The thalamus can synchronize activity across multiple nodes of cortical networks according to attention demands (Saalmann et al. 2012) and is a component of a tightly connected “central core” of brain regions engaged by multiple tasks and behaviors (Modha and Singh 2010). As it simultaneously modulates activity in cortical regions, the thalamus constitutes a set of key connector hubs for cortical networks. Improved knowledge of the spatial layout of those thalamic hubs, and the functional networks into which they are integrated and modulate, would influence models of human cortical function.

However, the structure and function of *human* thalamus has been an area of relatively limited focus, largely overshadowed by ongoing efforts committed to the study of cerebral cortex. In studies of brain networks, difficulty in discerning the multinuclear structure of the thalamus in noninvasive human MRI may have been linked to a tendency to regard it as a uniform unitary entity (He et al. 2009) or to neglect it entirely (Hagmann et al. 2008). However, the last 2 decades have seen a rapid advance in understanding thalamic function. Its clearest role, as a “relay” of sensory information from peripheral receptors to neocortex, is more complex, allowing incoming sensory information to be modulated or altered by ongoing activity in other brain networks (Sherman and Guillery 2002). Some components of the thalamus act as so-called first-order relays, conveying information from peripheral sensation or other brain regions, such as cerebellum and mammillary bodies; the largest part of thalamus consists of second-order relays, involved in conveying corticocortical information via a transthalamic route (Guillery 1995). Corticothalamocortical information flow may be modulated in the thalamus by the activity of other brain networks in a manner similar to modulation in first-order relays (Sherman and Guillery 2011). In this way, the thalamus plays a key role in corticocortical information flow and in modulating the activity of cortical networks (Theyel et al. 2010; Saalmann and Kastner 2011).

The multinuclear structure of the human thalamus itself has been mapped postmortem using selective histological staining (Morel et al. 1997; Schaltenbrand et al. 1977). In vivo thalamic subregions derived from diffusion tensor imaging (DTI) tractography-based segmentations have been regarded as directly equivalent to these nuclei, or sets of neighboring nuclei (Behrens, Johansen-Berg et al. 2003; Johansen-Berg et al. 2005). However, in nonhuman primates and mammals, there is reasonably strong evidence that the structural connectivity of the thalamus does not neatly map in this way. An early, data-driven, approach to determining the pattern of thalamocortical connectivity used retrograde labeling of thalamic nerve terminals in monkey frontal cortex (Kievit and Kuypers 1977) but found that thalamic regions with similar cortical connections were arranged in longitudinal “bands,” connecting to transverse cortical bands. These thalamic bands arranged medial-to-lateral mapping sequentially to rostral-to-caudal cortical bands, encompassing multiple distinct thalamic nuclei. This pattern was confirmed in follow-up studies (Royce 1983; Yeterian and Pandya 1985; Rouiller et al. 1999; Parvizi et al. 2006). More recently, using anterograde tracing across the entire mouse thalamus (Hunnicutt et al. 2014), it was demonstrated that anatomical connectivity-defined parcellations of the thalamus do not cleanly map onto histologically defined thalamic nuclei, instead encompassing multiple nuclei and even multiple nuclear groups. In humans, using an in vivo approach to human thalamic segmentation based on tissue signal in quantitative *T*_1_ and *T*_2_ magnetic resonance relaxation maps, we derived a thalamic segmentation qualitatively similar to histological atlases, but further showed that DTI-based segmentation of thalamus spanned across multiple “nuclei” in the same subjects (Traynor et al. 2011). Moreover, work interrogating the in vivo functional connectivity of the thalamus using functional MRI (Zhang et al. 2008, 2010) has indicated that thalamic regions are connected to more extensive cortical *networks*, reinforcing work in primates (Saalmann et al. 2012). In this context, the assumption that individual nuclei connect structurally to specific cortical regions probably cannot hold.

The relevance of thalamic structures to higher order cortical networks and especially cognition is reinforced by recent in vivo clinical studies on focal thalamic lesions. Indeed, most cortical lesion syndromes can be mimicked by regional thalamic injuries (Carrera and Bogousslavsky 2006). Interrogating such case studies, Krause et al. (2012) were able to coarsely segment subcortical patterns of known frontal-striatal-thalamic loops (see Alexander and Crutcher 1990) based on behavioral impairment in patients with spatially varied subcortical injuries. Focusing more directly on connectivity, Serra et al. (2013) further investigated 2 case studies of focal thalamic lesions, indicating that the very different behavioral profiles in 2 thalamic stroke patients may be explained by their different structural connectivity profiles to the rest of the brain. Lesion studies represent a blunt example, but these associations between regional structure and cognitive or behavioral function can also be detected in healthy populations. Using a similar diffusion connectivity approach, Philp et al. (2014) demonstrated that white matter indices along specific thalamocortical bundles relate to individual differences in expected domains, for example, radial diffusivity along the bundle from thalamus to precentral was significantly associated with motor function.

In combination, these studies implicate the connectional and structural architecture of the thalamus in wider cortical and subcortical networks that have clear cognitive and functional relevance. In this study, we explicitly examine how thalamic structural connectivity determines its integration into functional whole-brain networks. In place of fixed cortical regions of interest to define targets of thalamic connectivity, we use a data-driven technique to parcellate based on whole-brain structural connectivity (O'Muircheartaigh et al. 2011) using multimodal MRI data from a large open-access community sample of healthy participants (Nooner et al. 2012). Using the resulting partitions to drive functional connectivity analysis, we demonstrate the striking functional specificity of this structural parcellation. Further, we show that these functional networks can be reduced into 7 spatial patterns, represented prominently in 7 rostro-to-caudal cortical and subcortical networks connected with 7 medial-to-lateral thalamic regions. Demonstrated here in humans, this relatively simple architecture of whole-brain thalamocortical networks is strongly reminiscent of that described in both cat (Scannell et al. 1999) and monkey (Kievit and Kuypers 1977).

## Materials and Methods

### Dataset and Demographics

The dataset used here comes from the Nathan Kline Institute (NKI-Rockland sample) and has been released as part of the International Neuroimaging Data-sharing Initiative [http://fcon_1000.projects.nitrc.org/ (Nooner et al. 2012)]. Data were collected from a wide, community based, sample ranging in age from 6 to 85 years (mean 34.93, standard deviation 19.89). The procedures for the collection and sharing of data were acquired from the NKI review board, and written informed consent was acquired from each participant. Raw MRI datasets for the entire sample of 209 subjects available were initially downloaded, although 107 were later excluded due to inclusion and exclusion criteria (see below).

### Data Inclusion and Exclusion Factors

Previous work has demonstrated changing functional connectivity during childhood (Fair et al. 2010) and changes (reductions) in brain volume are largely linear with age between 18 and 65 years (Scahill et al. 2003), so this analysis was restricted to participants aged 18–65 years old. These age criteria led to an initial exclusion of 71 datasets. This study made use of 3 of the available MRI modalities from each dataset. For all subjects, a *T*_1_-weighted image, a resting-state fMRI time series and a diffusion-weighted dataset were required in order to undertake the planned analyses. Any dataset without all 3 of these was therefore excluded (4 excluded). Finally, the fMRI data were assessed based on interscan framewise displacement, revealed by alignment of the time series. Subjects with more than 25 spikes of interscan displacement >0.2 mm on fMRI (i.e., 10% of all timepoints) were also excluded. This led to a further 32 datasets being excluded from further analysis. The final resulting sample size was 102 (40 female, mean age 33.9 years, standard deviation 12.9 years). Dataset IDs used in the final analysis are listed in Supplementary Table 1.

### Magnetic Resonance Imaging Parameters

All data were collected on a single 3-Tesla Siemens TrioTim MRI scanner. For anatomical localization and spatial normalization a *T*_1_-weighted magnetization prepared rapid gradient echo (MPRAGE) structural image was acquired with a TR/TE of 2500 ms/3.5 ms, inversion time of 1200 ms, flip angle of 8°, and a field of view of 256 mm × 256 mm over 192 slices resulting in an isotropic resolution of 1 mm. For functional MRI acquisition, a gradient echo-planar imaging (EPI) sequence was used. For each of 260 acquisitions, fMRI data were collected with a TR/TE of 2500 ms/30 ms, flip angle of 80°, 38 slices collected with a field of view of 216 mm × 216 mm leading to an eventual isotropic resolution of 3 mm. Diffusion data were collected with a spin-echo EPI sequence and a TR/TE of 10 000 ms/91 ms, field of view of 256 mm × 256 mm over 58 slices with an isotropic resolution of 2 mm. In total, 76 volumes were collected, 64 of which had a diffusion weighting of 1000 s mm^−2^ and the other 12 were acquired without any diffusion weighting.

### Data Preprocessing

Diffusion-weighted images were corrected for the effects of distortion induced by eddy currents and interscan motion. Data were prepared for probabilistic tractography using bedpostX (Behrens, Woolrich et al. 2003). This method uses Monte Carlo Markov chain sampling to estimate the diffusion parameters for every voxel, calculating the necessary parameters for probabilistic tractography. We modeled for up to 2 possible fiber populations per voxel and used a burn-in of 5000 to ensure the convergence of the Markov chains (Behrens et al. 2007). The initial image without diffusion weighting (i.e., approximately *T*_2_ weighted) was rigidly aligned to the anatomical *T*_1_-weighted image. The *T*_1_-weighted image was nonlinearly registered to the MNI *T*_1_-weighted template using fnirt.

Data preprocessing for functional data comprised 3 steps designed to minimize the number of interpolations. Correction for slice timing differences induced by the interleaved fMRI acquisition and correction of the time series for between-scan head movements were performed concurrently using a 4D correction algorithm (FmriRealign4D, (Roche 2011) distributed as part of the nipy package). Data were temporally high-pass filtered at 0.01 Hz using fslmaths. Realignment parameters for every subject were recorded and expanded (Friston et al. 1996) and interscan motion of more than 0.2 mm was recorded to include as scan nulling regressors later on (Lemieux et al. 2007; Power et al. 2013). Finally, data were rigidly linearly coregistered to the *T*_1_-weighted image using boundary-based registration (Greve and Fischl 2009). Functional data were resampled to 2-mm isotropic resolution in MNI space using the nonlinear transformation between the *T*_1_-weighted image and the MNI template.

### Diffusion Tensor Tractography

Left and right thalamic regions of interest, obtained from the Harvard Oxford atlas packaged in FSL, were used as seed regions for probabilistic tractography with no exclusion masks provided. For each subject, we initiated probabilistic tractography from each voxel in each thalamus separately, leading to 1465 volumes per subject for the left thalamus and 1514 volumes per subject for the right thalamus. Each volume represented the count of streamlines reaching every other voxel in the brain from this thalamic seed voxel from 5000 iterations. The resulting tractograms were resampled to MNI space at 2-mm isotropic resolution. These volumes were concatenated into a single 4D volume. This resulting image was smoothed using a 4-mm full-width half-maximum Gaussian kernel in seed space, that is, the tractogram created from each voxel became a Gaussian-weighted average of the tractograms initiated from surrounding thalamic seeds.

These 4D volumes, for each hemisphere separately, were fed into a group independent component analysis (ICA) using tensor ICA (Beckmann and Smith 2005). The number of components was estimated automatically. Resulting components represent patterns of tractography-defined connectivity that are consistent in their thalamic origin across subjects. Using this output from melodic and tools provided in the nipy suite (Millman and Brett 2007), the weighted thalamic origin of each spatial component was mapped back onto the thalamus in MNI space. Spatial components are displayed after thresholding using mixture modelling (Beckmann et al. 2003) as in O'Muircheartaigh et al. (2011). Their thalamic origin was thresholded at a normalized Z-value of 2.3, illustrating thalamic regions that are represented by the spatial component.

### Functional Connectivity Analysis

The thalamic origin maps identified from the thalamic tractography data were used in a spatial regression on the spatially normalized and unsmoothed fMRI data. For every subject, a functional time series was calculated for the weighted thalamic origin of each spatial independent component (fsl_glm). As confounds, average white matter and cerebro-spinal fluid (CSF) signal were calculated from masks with a conservative threshold (50% for CSF and 75% for white matter). These masks were conservative to limit the risk of inadvertently modeling the global signal (for further discussion see (Saad et al. 2012). A design matrix was created incorporating the set of thalamic time series, expanded motion parameters, scans with excessive motion recorded during motion correction, average white matter and average CSF signal. This design was input into a general linear model for every subject (fsl_glm). These steps are analogous to the dual regression technique commonly used in resting-state network analysis (Zuo et al. 2010), but with the initial regression being the thalamic origin maps calculated from the tractography and ICA pipeline in place of ICA-derived resting-state networks. This model was tested for left and right thalamus separately.

The resulting individual connectivity maps were concatenated across subjects, spatially smoothed by a 6-mm FWHM Gaussian kernel, and group average functional connectivity was calculated by permutation (randomise), with age and gender modeled as covariates of no interest. This group analysis was performed separately for every thalamic subregion identified in the ICA analysis, with a correction for multiple comparisons being performed voxelwise, using threshold free cluster enhancement (TFCE, (Smith and Nichols 2009)).

### Clustering of Thalamic Functional Connectivity Maps

**Figure 1.**
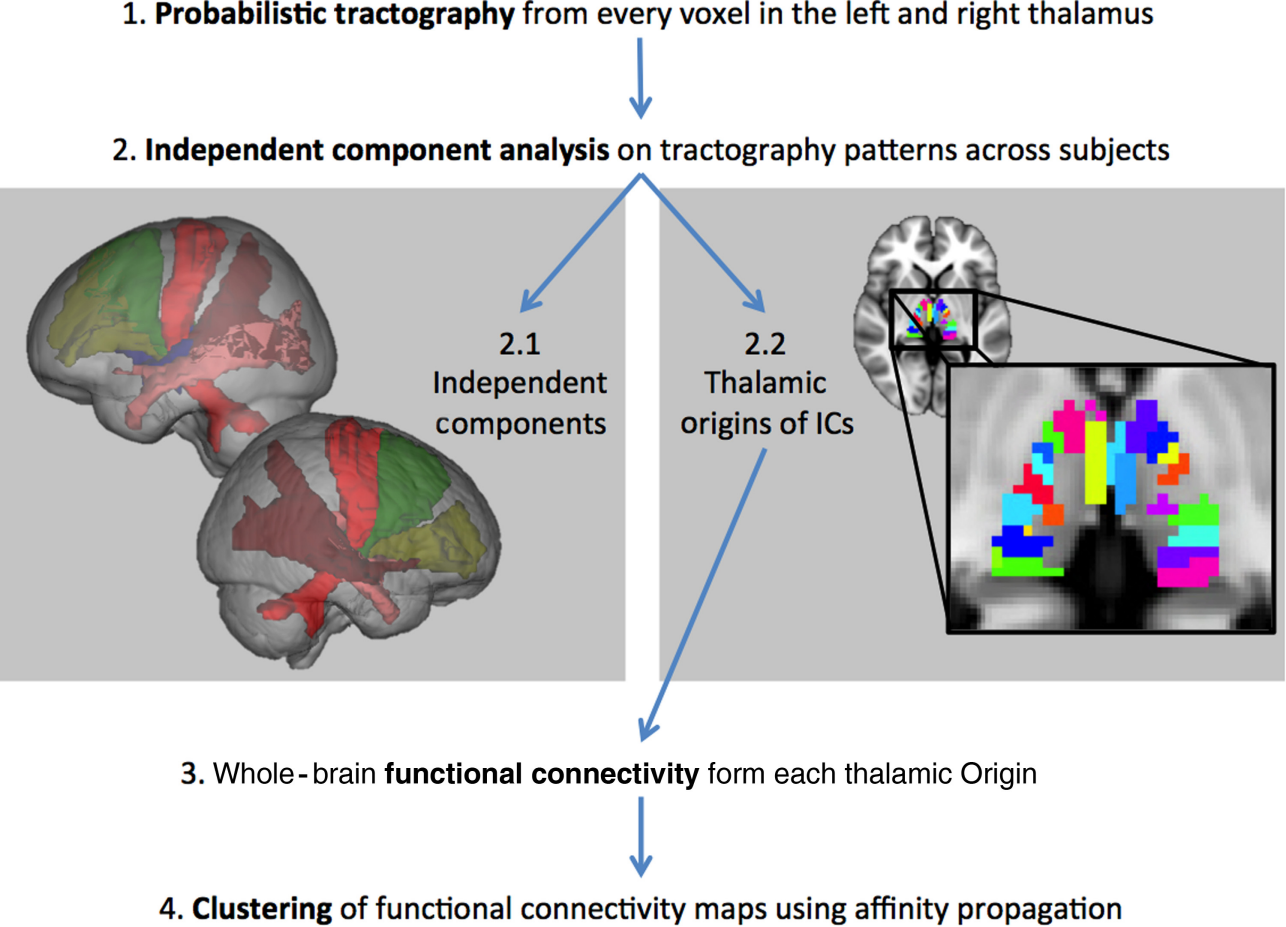
Basic workflow showing the main analysis steps in this experiment. After image preprocessing including spatial registration, probabilistic tractography is performed from every voxel in the thalamus for every subject (1). The resulting set of tractograms are input into a spatial tensor independent component analysis (2) resulting in spatial components that represent consistent patterns of white matter connectivity (2.1, with representative example bundles) and the regions of the thalamus from which the originate (2.2). These thalamic regions are used as seeds in whole-brain functional connectivity analyses (3) and the resulting patterns are clustered using affinity propogation to identify brain regions that show consistent connectivity with thalamic regions (4).

Finally, to investigate the similarity and possible redundancy of the resulting functional connectivity maps, a similarity matrix was constructed using the raw correlation between each of the functional connectivity t-maps. This similarity matrix was then clustered using affinity propagation (Frey and Dueck 2007), with median similarity of the functional connectivity maps to each other acting as the initial preference value. To illustrate regions of the brain driving the clustering solution, conjunction tests (Nichols et al. 2005) were performed across the functional connectivity maps for each cluster. This results in maps that demonstrate only voxels that show significant functional connectivity in *all* maps assigned to each cluster, and hence summarize the shared functional connectivity of a set of thalamic structural connections driving the clustering solution. Figure 1 illustrates a simple breakdown of the analysis pipeline.

## Results

Tensor ICA of the probabilistic diffusion tractography data resulted in 25 and 26 spatial components for the left and right thalamus, respectively, representing consistent patterns of DTI tractography-based structural connectivity from the thalamus. Using the same output, the spatial origin of these components was back-reconstructed onto the thalamus, revealing the specific thalamic origins for these presumed white matter bundles (see Fig. 1 right for representative connectivity-defined thalamic regions and connectivity patterns).

**Figure 2.**
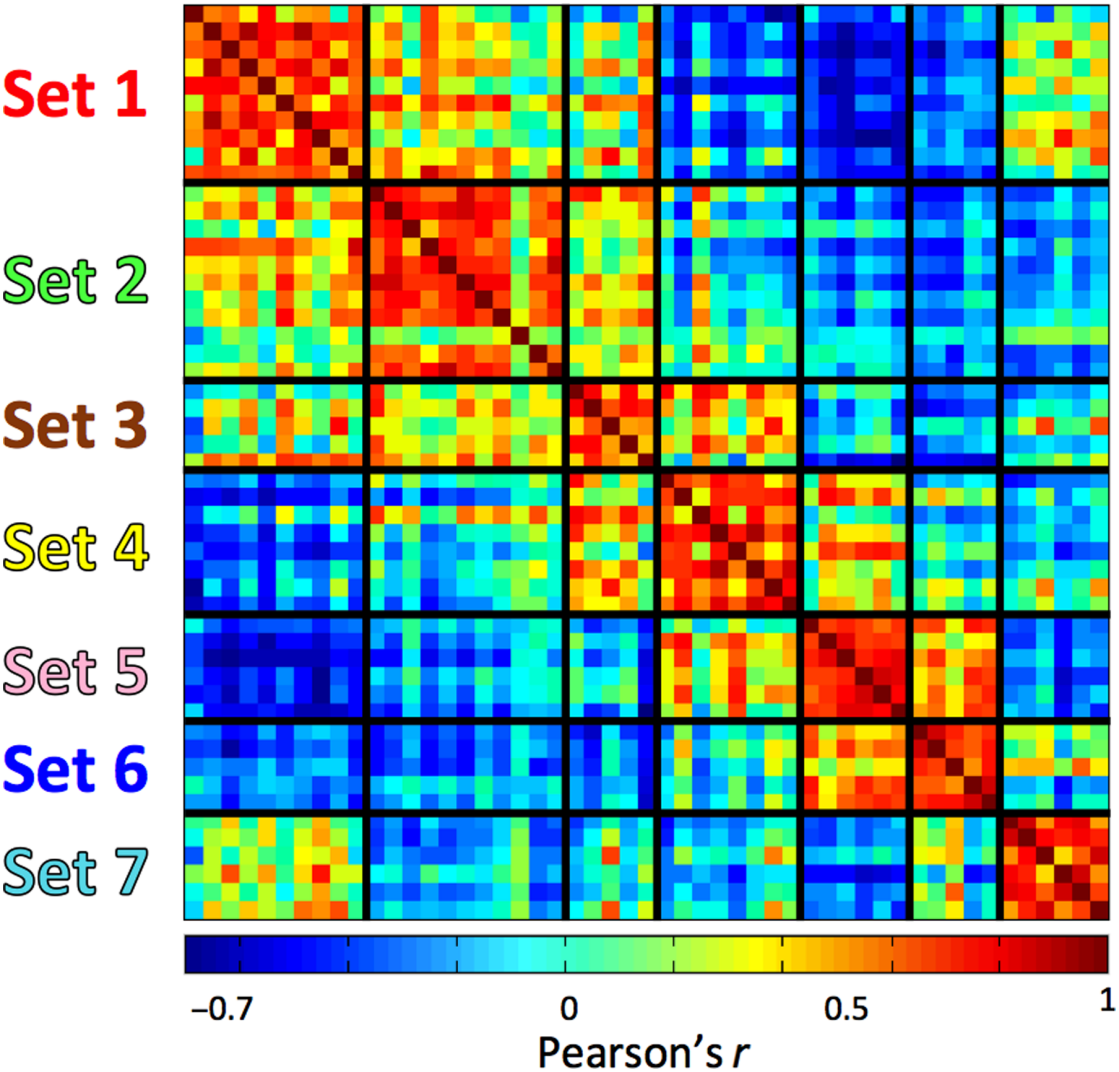
Correlation matrix between spatial functional connectivity networks for each thalamic region identified using DTI connectivity. Note there are both strong positive and negative correlations between these networks. Deep black lines indicate the clustering solution achieved by affinity propagation, reducing the 51 thalamic regions to 7 bilateral sets (labeled on the *Y*-axis).

Using resting-state fMRI in the same subjects, whole-brain functional connectivity with these thalamic seed regions resulted in highly significant (*P* < 0.05, corrected for multiple comparisons using TFCE) and anatomically specific functional connectivity in cortical and subcortical regions. The results of the affinity propagation cluster analysis on the functional connectivity maps (see correlogram, Fig. 2) indicated 7 major sets of functional networks representing between 5 and 11 thalamic regions per set, which we have labeled in an order from anterior to posterior, following their major functional correlates in midline structures.

**Figure 3.**
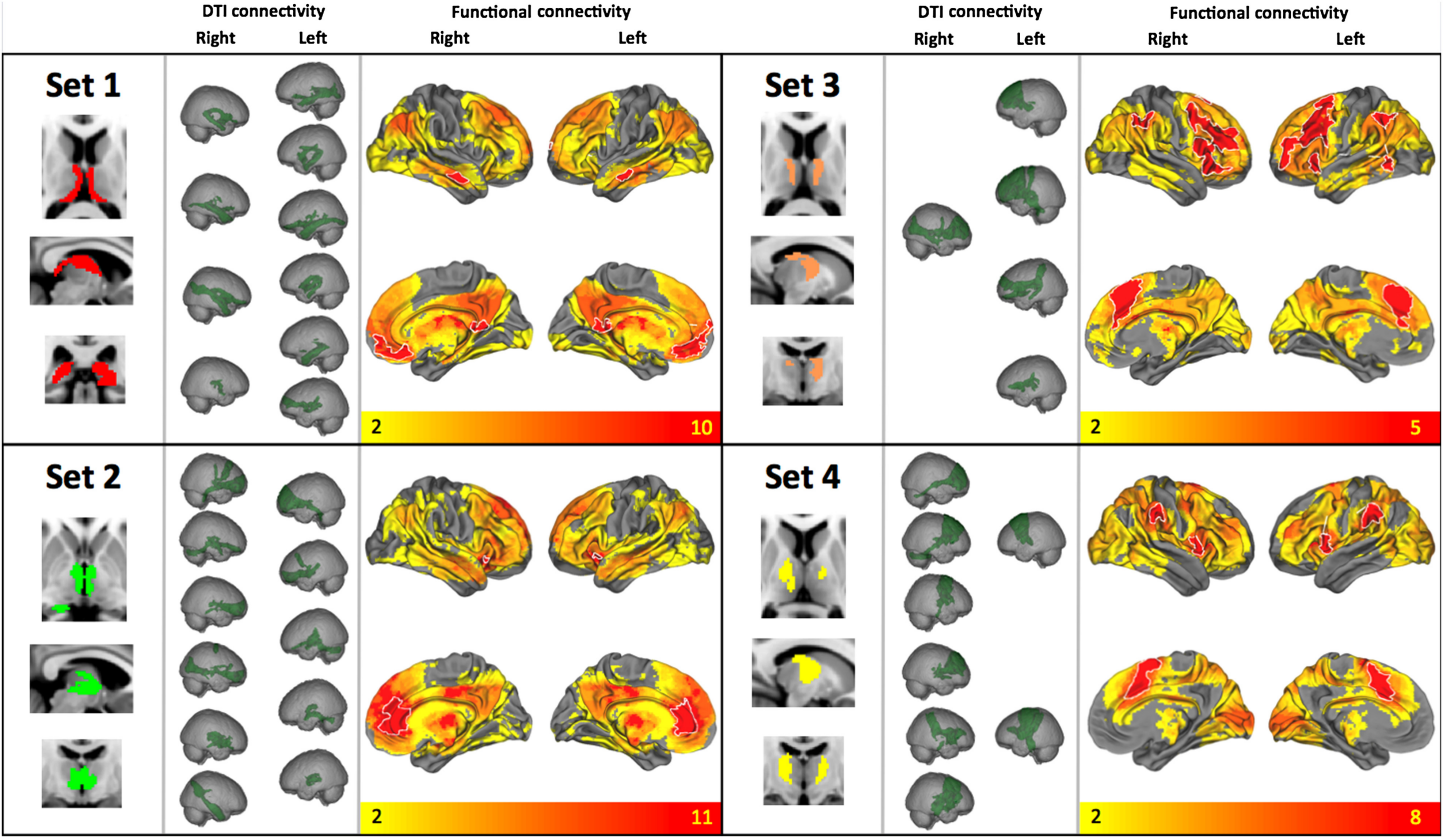
Features of the sets of functional networks in terms of their thalamic origin (left), tractography-defined connectivity (middle), and functional connectivity (overlap across all images assigned to the set in the clustering solution, right). Top left, “set 1”: The most medial cluster of thalamic components were functionally connected with medial orbitofrontal cortex, bilateral middle temporal gyrus and bilateral hippocampus, as well as posterior cingulate and retrosplenial cortex posteriorly. The resulting tractograms resembled aspects of the fornix as well as simple thalamofrontal bundles. Bottom left, “set 2”: This cluster of thalamic regions, more lateral to the first, showed functional connectivity with ventral anterior cingulate, ventral caudate and anterior putamen. Top right, “set 3”: The third cluster was functionally connected to a bilateral frontoparietal network including middle frontal gyrus (encompassing Broca's area), ventral frontal opercula, and bilateral posterior supramarginal gyrus. In addition, midline structures including anterior cingulate and mesial aspects of the superior frontal gyrus. This set was predominantly left lateralized and showed DTI connectivity to middle and superior aspects of the frontal lobe. Bottom right, “set 4”: Overlapping in functional connectivity with cluster 3, cluster 4 showed functional connectivity with bilateral insula, premotor and supplementary motor cortex, as well as bilateral anterior supramarginal gyrus. The thalamic regions contributing to this cluster were predominantly right hemisphere and spatially overlapping with those in the left hemisphere in cluster 3.

**Figure 4.**
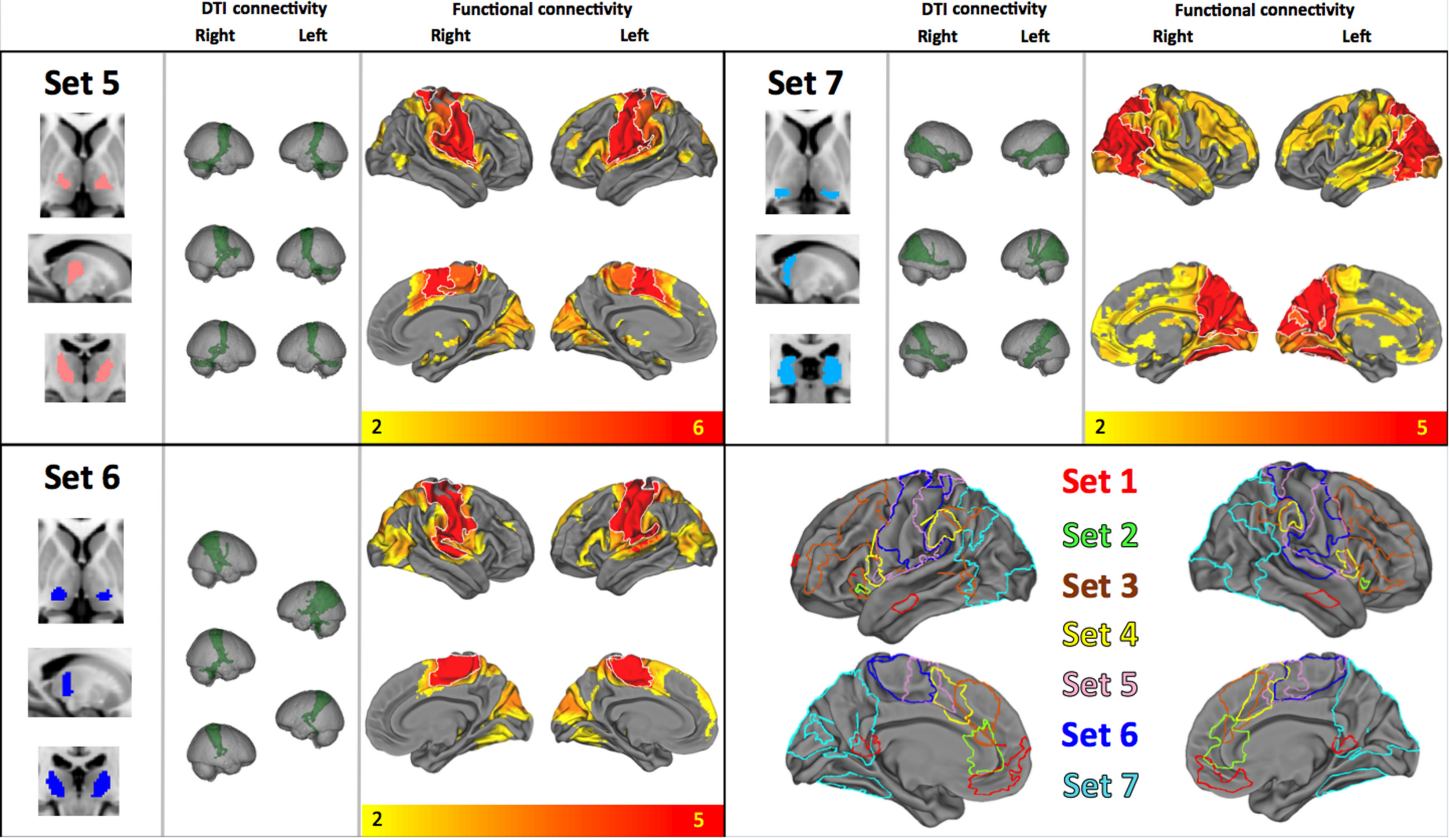
Top left, “set 5”: This cluster showed functional connectivity along the entire precentral gyrus bilaterally, supplementary motor cortex, as well as posterior and ventral putamen and lobes V and VI of the cerebellum. DTI connectivity mirrored this, indicating connections to motor cortex, supplementary motor area (SMA), and cerebellum. Bottom left, “set 6”: This cluster shows functional connectivity to postcentral gyri and some precentral. Mirroring this, most tractography bundles connected with cortex only. Top right, “set 7”: This posterior cluster shows overlap with the expected position of the pulvinar and shows structural and functional connections to posterior parietal and occipital regions including visual cortex, visual association areas, ventral temporal cortices including fusiform and parahippocampal gyri but no subcortical or cerebellar areas. DTI connectivity data show posterior projections to superior occipital and posterior parietal regions. Bottom right: Summary of the areas of cortical functional connectivity with their diffusion tractography-derived seed regions, displayed in a rostral to caudal axis and in medial to surface bands in the thalamus.

The underlying thalamic origin of these sets demonstrated a pattern of medial-to-lateral bands, eminating radially from the mesial aspect of each thalamus (see Figs 3 and 4). The only exception to this pattern was the limbic group, which followed the trajectory of the fornix (posterior to anterior, curved around the medial and dorsal aspect of the thalamus, see Fig. 3, set 1). The patterns were largely symmetric with the exception of sets 3 and 4 (Fig. 3).

**Figure 5.**
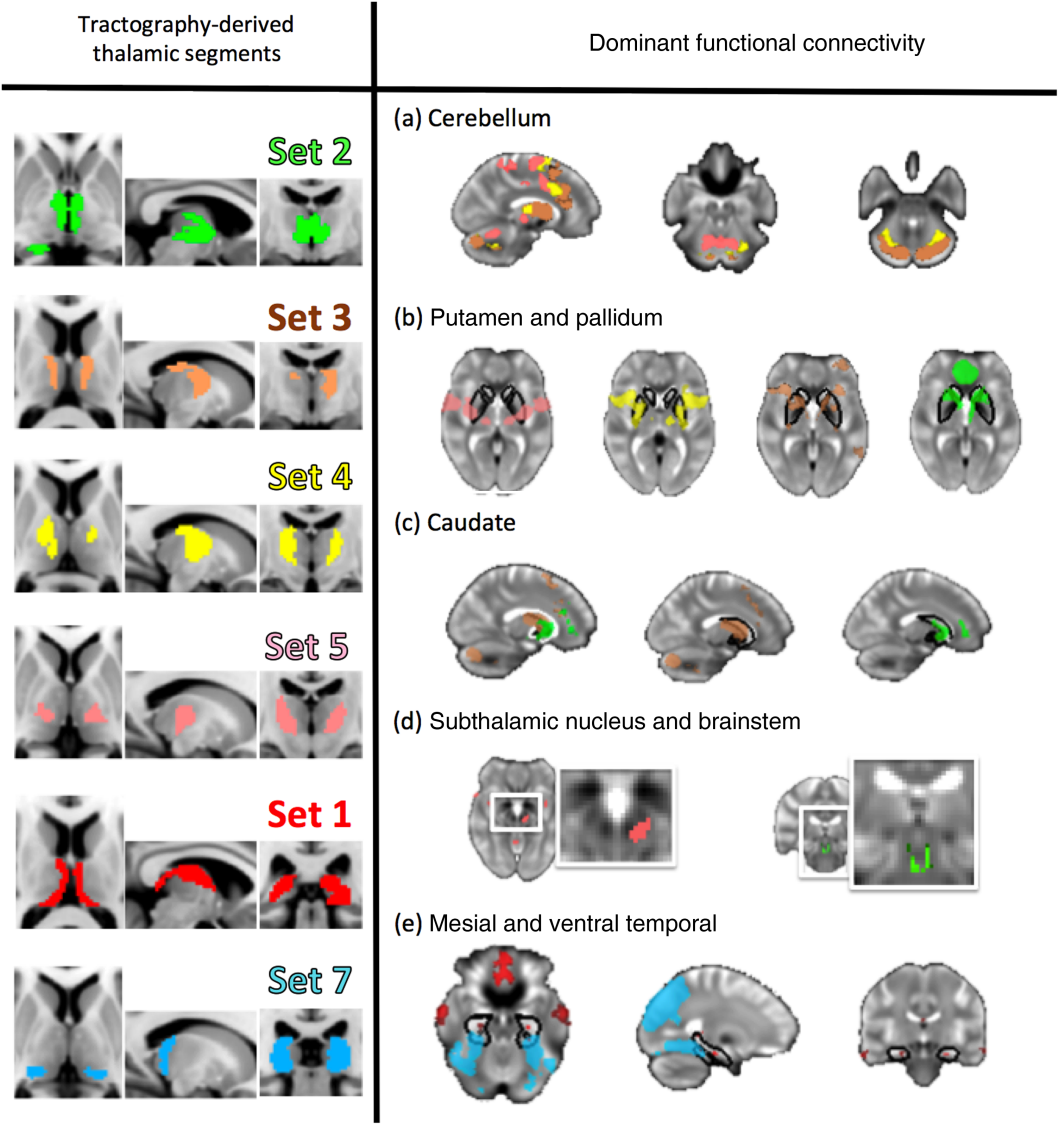
Specific thalamic regions map onto specific subcortical and mesial temporal lobe subregions. Anterior and ventrolateral thalamic sets especially map onto specific known thalamocortico-basal-ganglial loops showing differential connectivity in cerebellar (a), striatal (b, c), and subthalamic/brain stem regions (d). These regions included subthalamic and pedunculopontine nuclei (d, left and right, respectively). Medial thalamus (set 1) and posterior sets (set 7) correspond to limbic and ventral temporal association areas (e).

**Figure 6.**
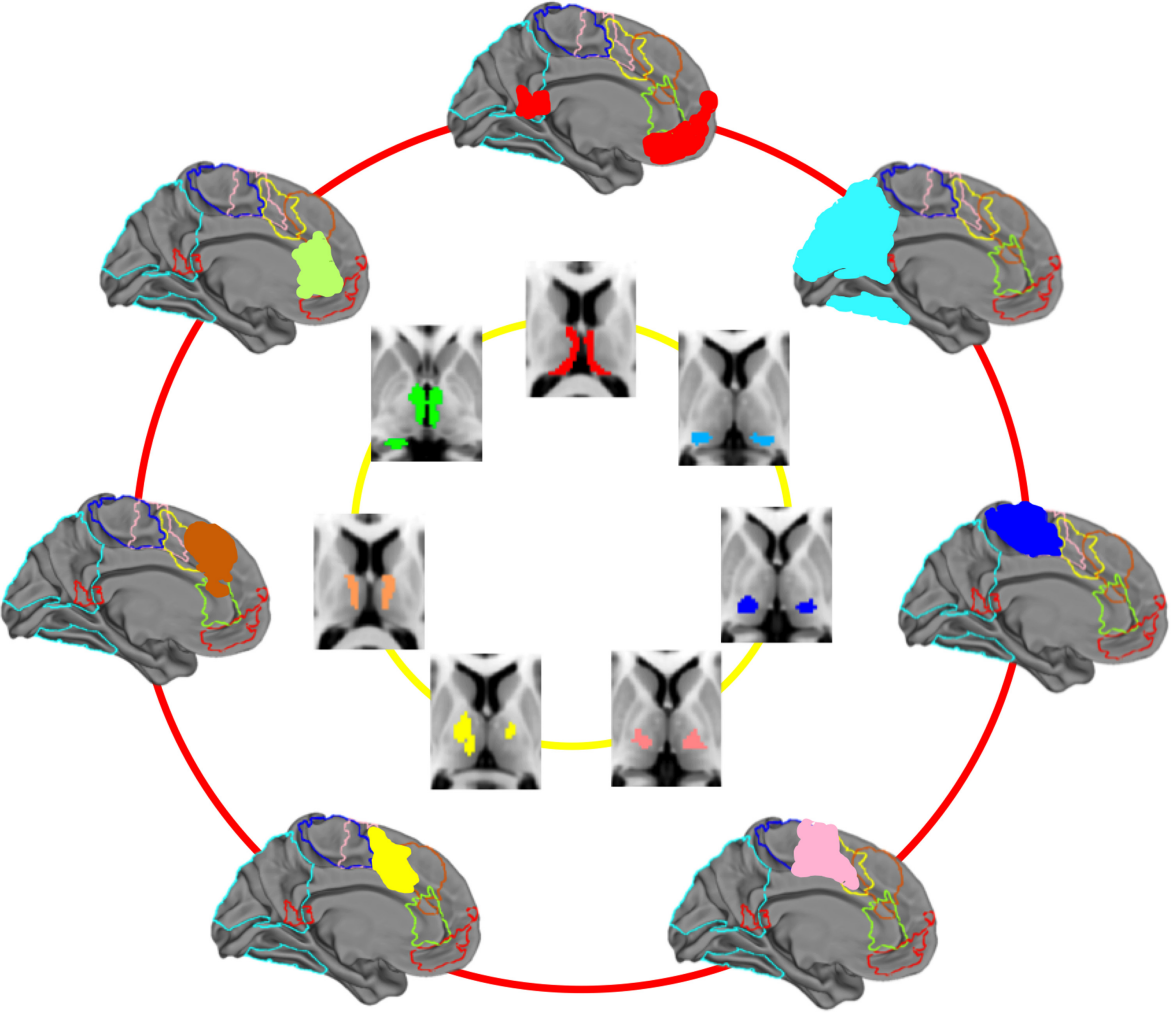
Summary of the rostral-caudal pattern of cortical connectivity demonstrating specific medial to lateral bands in the thalamus.

Overlap maps and formal conjunction tests (indicated with a white outline) on the resting-state connectivity maps across the sets are also shown in Figures 3 and 4, thresholded at *P* < 0.05 corrected for multiple comparisons using TFCE. These conjunction results summarize the cortical regions which have shared functional connectivity with all of the thalamic regions in a set (see Supplementary Table 2 for a description of these regions). These shared regions of connectivity show a distinct rostro-caudal profile across the cortical midline that maps onto the anteromedial-posterolateral structural connectivity profile in the thalamus. These are color coded per set in Figure 6 to illustrate differing midline cortical connectivity and in Figure 5 to illustrate differential connectivity in subcortical and cerebellar regions of the brain.

For completeness, we include a group average fMRI image in MNI space (Supplementary Image 1), the raw images of the 51 independent components (Supplementary Image 2), their thalamic origins (Supplementary Image 3) and their functional connectivity (TFCE-corrected *P*-value maps—Supplementary Image 4; raw T-maps—Supplementary Image 5), and the clustering solution (Supplementary Table 3).

## Discussion

We demonstrate here the architecture of structural connectivity of the entire human thalamus, using an unbiased, data-driven approach to DTI which allowed thalamus to be parcellated blindly on the basis of its structural connections. We show that the each resulting thalamic parcel has a specific pattern of functional connectivity with a *distributed* network of cortical and subcortical regions, and negative connectivity with activity in competing networks (Fig. 1). Further, we indicate that the 51 thalamocortical functional networks identified in this work could be reduced to 7 sets of networks with very similar anatomical features within each set but minimal anatomical overlap between sets; each set is composed of mostly neighboring thalamic segments. These sets of neighboring thalamic segments were distributed in bands which have a distinct orientation and are arranged sequentially from medio-superio-anterior to latero-infero-posterior. Neighboring thalamic regions were especially distinctive with regards to their functional connectivity to subcortical, cerebellar, and mesial temporal structures (see Fig. 5). Thalamic segments have shared cortical connectivity particularly with medial cortical regions; the medio-superio-anterior to latero-infero-posterior arrangement of thalamic segments mapped to caudal to rostral regions of medial cortex in keeping with their tractography-derived structural connectivity.

These functional networks are distinctly different from networks derived from an ICA of resting-state fMRI data alone. Most functional networks involve more than just their tractography-derived thalamocortical target, further recruiting other cortical and subcortical regions. We propose that our findings demonstrate the underlying architecture of large-scale thalamocortical systems, and provide evidence of an anatomical basis for thalamic selection between competing cortical networks via specific cortical nodes. Cat corticothalamic connection data suggest a relatively simple architecture consisting of 4 corticothalamic modules (Scannell et al. 1999). Our results support their conclusion that this “suggests that, in principle, relatively few sources of variability can account for much of the variability in the gross connection pattern of the thalamo-cortical network. We think this apparent simplicity shows that a small number of simple rules, reflecting genetic and/or developmental economy, have been favoured by evolution in the design of the thalamo-cortical network” (p. 292).

Identifying a distinct set of functional networks has apparent analogies with identifying functional networks using ICA in resting-state fMRI data. The networks demonstrated here are distinctly different from the typical functional networks identified using ICA in resting-state fMRI data (Damoiseaux et al. 2006; Fox and Raichle 2007; Cole et al. 2010). Typically, such an approach identifies networks which are anatomically more restricted than those shown here: for example, typical resting-state networks might consist of striate regions, or extrastriate regions, or auditory regions, or a limited set of sensorimotor regions, or frontoparietal attention regions within one hemisphere, or the so-called default-mode network. Such restricted regions and networks show very strong synchronization of activity within the network, and may be key modules of larger functional networks, but may have limited correspondence with the architecture of thalamocortical networks. As is increasingly clear (Calhoun et al. 2012; Smith et al. 2012), it is difficult to conceive that a single network identified using ICA in resting-state fMRI data could reflect a single distinct mode of large-scale brain function; in contrast, we speculate that the thalamocortical networks may constitute distinct, and anatomically constrained, brain modes or brain states. Resting-state networks identified using ICA of fMRI may represent just the most closely synchronized neighboring nodes of larger networks; as such they may reflect tightly coupled subnetworks of key importance to brain function, but their inputs and relationships to other networks are less clear. The networks identified in this study are substantially different, revealing the temporally correlated, and anticorrelated, thalamocortical networks coupled with specific thalamocortical inputs. Our findings implicate specific thalamic regions and their structural connections in mediating the process of brain network selection.

The thalamocortical structural connections demonstrated constitute the principal *gross* connections between each thalamic segment and other brain regions. We speculate that the structural connections between each thalamic segment and a limited region of cortex subserves the influence of that thalamic segment on wider cortical networks—in other words, the thalamus modulates large-scale cortical networks through its connectivity with one cortical node or a small neighboring set of cortical nodes. Nonetheless, we recognize the limitations of our DTI approach, and its insensitivity especially to sparse connections between brain regions. We note that the functionally connected networks are anatomically in good correspondence with the full range of cortical connections with thalamic regions demonstrated in animal studies and, hence, the structural connections we parcellate may be merely the strongest of a much more anatomically widespread set of connections.

### Anatomical Features

Anatomically, the resulting functional/structural parcellation shows good comparability with animal work. This first cluster of thalamic regions (Fig. 3, set 1) appears to overlap primarily with anterior nuclei, midline nuclei, and medial pulvinar. The functional connections of this cluster are in keeping with the known connections found in monkeys. In the macaque, the subiculum projects to anterior nucleus, reuniens nucleus and medial pulvinar, and entorhinal cortex projects to medial pulvinar (Aggleton et al. 1986). These thalamic regions in the macaque also project to posteromedial cortex: posterior thalamus has connections to medial parietal and posterior cingulate cortex (Parvizi et al. 2006), and anterior nucleus projects to retrosplenial cortex in the rat (Garden et al. 2009).

The second cluster of thalamic regions (set 2) appears to overlap primarily the medial nuclear group in addition to anterior regions. In rhesus monkeys, mediodorsal nucleus connects to polar anterior prefrontal cortex, ventromedial prefrontal cortex, anterior cingulate, and insula (Bachevalier et al. 1997); the medial (magnocellular) part of mediodorsal nucleus projects to more ventral and medial cortex, whereas the lateral (parvicellular) part projects more dorsally and laterally (Giguere and Goldman-Rakic 1988). In cynomolgus and macaque monkeys, connections more posteriorly in frontal lobe, to pre-supplementary motor area (preSMA) and premotor regions, have also been noted (Erickson and Lewis 2004; Morel et al. 2005). Cortex at the temporal pole is also connected to mediodorsal nucleus (Gower 1989). This cluster also includes functional connectivity with medial parietal and retrosplenial cortex, which in the macaque connect with anterior thalamus (Parvizi et al. 2006).

Ventral anterior and ventral lateral nuclear regions appear to overlap with the third and fourth thalamic cluster (sets 3 and 4, Fig. 3). In the macaque and in cats, these nuclei include projections to premotor cortex and SMA (Royce 1983; Rouiller et al. 1999; Morel et al. 2005). Set 3 showed a leftward asymmetry and set 4 a rightward asymmetry. This may indicate relatively lateralized representations of their respective functional correlates, for example, the third set includes Broca's area. However, this may be wishful thinking and it may simply be an arbitrary split of a large similar cluster into 2 distinct sets (see Fig. 2).

Sets 5 and 6 (Fig. 4) appears to overlap ventral lateral and ventroposterior nuclear regions. In the macaque, the ventroposterolateral (VPL) nucleus projects to primary motor and premotor cortices (Rouiller et al. 1999; Morel et al. 2005). In rhesus and other monkey species, ventroposterior cortex connects to insula (Burton and Jones 1976; Mufson and Mesulam 1984). Corticothalamic connections have been demonstrated in the rhesus monkey between posterior parietal cortex and ventral lateral, ventroposterior and lateral posterior nuclear regions (Yeterian and Pandya 1985), overlapping with regions seen here; with rostral-superior to caudal-inferior cortical regions connected to these nuclei in an anterior-posterior arrangement.

Set 7 appears to overlap with lateral posterior nucleus and, most probably, the pulvinar. In the rat, this region is extensively connected with medial and lateral parietal and occipital cortex (Kamishina et al. 2009). Connections in monkeys have also been noted to posterior parietal (Yeterian and Pandya 1985) and insular (Burton and Jones 1976) regions. Connections with frontal eye fields have not been noted specifically from this nucleus, but from nearby ventral lateral, parafascicular, and medial pulvinar regions (Huerta et al. 1987). Overlap with the pulvinar is presumably mostly with medial pulvinar since this is substantially bigger than lateral pulvinar in rhesus monkey and man (Romanski et al. 1997). The lateral pulvinar connects to an extended region of the lateral temporal cortex stretching down to the temporal pole. Although the lateral pulvinar is certainly represented in this parcellation of the thalamus itself, this set appears to be dominated by functional connectivity more representative of the medial pulvinar. The medial pulvinar has connections with inferior temporal (Benevento and Rezak 1976) and superior temporal cortex (Romanski et al. 1997) but also inferior parietal lobule (Divac et al. 1977), dorsolateral and dorsomedial frontal cortex, orbital cortex and insula (Romanski et al. 1997), as well as striate and extrastriate regions (Adams et al. 2000; Grieve et al. 2000). Corticothalamic connectivity from posterior cingulate and retrosplenial cortex is relatively specific to the medial, as opposed to lateral or inferior, pulvinar (Baleydier and Mauguière 1987), and these are the cortical areas seen dominating functional coactivity in set 7.

The clustering solution provides very intriguing insights into relationships between functional networks. Figure 2 demonstrates that some thalamic clusters are both strongly functionally anticorrelated with each other and others (especially neighbors) positively correlated. Our data suggest that thalamocortical connectivity may provide a compelling mechanism behind the selection between competing functional and apparently cortical networks. In addition to casting light on fundamental network architecture of the brain, these results may reflect clinically relevant processes. The functional connectivity networks linked to each thalamic subregion correspond to deficits seen clinically in response to specific thalamic insults. Regions corresponding to the pulvinar (Fig. 4, set 7) elicit a visual attention network, including frontal eye fields, that are consistent with the spatial neglect that pulvinar injuries can induce (Karnath et al. 2002; Saalmann and Kastner 2011), just as regions of the medial thalamus underlying the fornix functionally co-activate with the hippocampus (Fig. 3, set 1) (Aggleton et al. 1986). There is also a negative correlation between thalamic networks co-activating with the parahippocampal gyrus and thalamic networks co-activating with the hippocampus itself, corresponding to distinctions in these networks observed by others (Ward et al. 2013).

Although previous work has investigated thalamic functional connectivity with the rest of the brain (e.g., Zhang et al. 2008), studies have tended to segment the thalamus based on its connectivity profile with respect to predefined targets of interest. However, network organization and the spatial and functional heterogeneity of the brain may make such approaches problematic. We have attempted to overcome such problems in the current study by informing our functional connectivity analyses based on structural connectivity anatomical information. The network correlates of thalamic connectivity clearly indicate specific anatomical profiles. The network overlap images (Figs 5 and 6) indicates a core set of cortical and subcortical regions, respectively, that show functional coherence with the thalamus, some of which are implicated in the default-mode network (posterior parietal, mesial frontal), the executive network (SMA, basal ganglia), memory (hippocampus, retrosplenial), and motor planning (basal ganglia). These regions are spatially distributed and overlapping. Clear in Figure 5, distinctive limbic, striatal, and cerebellar patterns of functional connectivity reflect the striatal thalamocortical loops described in Alexander and Crutcher (1990) and carefully delineated using diffusion MRI by Draganski et al. (2008).

Approaches integrating structural and functional connectivity could work toward elucidating specific dysfunctions in neurological and psychiatric disease. In psychiatry, Woodward et al. (2012), building on the work of Zhang et al. (2010) and using a seed-based approach, demonstrated increased functional connectivity between thalamus and somatosensory regions and decreased connectivity between anterior thalamus and prefrontal cortices in participants with schizophrenia or schizoaffective disorder compared with controls. More recently, this result has been independently replicated using a data-driven functional parcellation of the thalamus based on whole-brain functional connectivity (Anticevic et al. 2014). In addition, the result was extended to bipolar depression indicating that this thalamocortical change may be more indicative of common symptomology than exact diagnosis. As a whole structure however, a recent meta-analytic approach found the thalamus (as a whole) to be generally altered across a range of different brain disorders (Crossley et al. 2014).

Similarly, changes in the thalamus are a core feature of many neurological disorders. Focusing on epilepsy, we have used connectional patterns of the thalamus to delineate a functionally meaningful abnormal network in juvenile myoclonic epilepsy (O'Muircheartaigh et al. 2012), indicating changes in a very specific and local thalamocortical circuit, between the anterior thalamus and mesial frontal cortex. This was evident using both structural and functional imaging metrics, and replicated thalamic changes from earlier work focusing on white matter in a similar group (Deppe et al. 2008). Changes in the thalamus are specific to the type of epilepsy, with mesial temporal lobe epilepsy showing a different pattern of thalamic changes centered on the mediodorsal regions (Bernhardt et al. 2012; Barron et al. 2013; Keller et al. 2014) appropriate to the likely epileptogenic network.

In this way, physiologically motivated models of thalamocortical networks are relevant to understand transitions to epileptic seizure dynamics (Rodrigues et al. 2009). However, such work tends to model thalamus and cortex as single regions. The results provided here could provide a more anatomically constrained and realistic approach to understand whole-brain dynamics, integrating connectivity data with theoretical models of dynamics at networks nodes (themselves sets of cortical or thalamic regions) and better reflecting the reality of the underlying anatomy and pathology. For example, we have demonstrated abnormal network topology in idiopathic generalized epilepsy using EEG (Chowdhury et al. 2014), itself predictable from theoretical modeling on the interactions between network topology and local node dynamics in creating dynamic transitions to different networks states, including seizure-like activity (Terry et al. 2012), and used this empirical data to update models of mechanisms of seizure onset (Petkov et al. 2014). We anticipate that this approach to understanding whole-brain dynamics, which combines connectivity data with theoretical models of dynamics at the network nodes, will be usefully informed by a better account of the distribution of thalamocortical connectivity which our work here provides.

### Methodological Considerations

As commented by others (e.g., Honey et al. 2009), although structural connectivity partially explains functional connectivity, the opposite is probably not true, at least as inferred by the temporal resolution currently possible using fMRI. Although tractography may give a good indication of zero-lag functional connections, later corticocortical propagation of functional activity is also evident here, and these different sources remain difficult to separate given the timescale of the BOLD signal. Greicius et al. (2009) demonstrated that the default-mode resting-state network (comprising mesial temporal lobe, posterior cingulate, and medial frontal cortex) was anatomically connected by different aspects of the cingulum bundle. They commented that although they were unable to identify any connections between the mesial temporal lobe and mesial frontal cortex, this might represent a false negative. However, this assumes that corticocortical connections alone underlie functional coherence in this network. Our results show both thalamotemporal and thalamofrontal connections correlating with activity in subtly different regions of what is classically called the default-mode network (sets 1, 2, and 7, Figs 3 and 4).

There are some clear limitations in how white matter tractography can be interpreted (Jones and Cercignani 2010). First, we cannot tell from where a track originates or where it terminates, whether a white matter bundle is mono- or multisynaptic, or whether the fiber is afferent or efferent (Jbabdi and Johansen-Berg 2011). In using tensor ICA (Beckmann and Smith 2005), we are also assuming that the pattern of structural connectivity is approximately consistent across the population. In the context of the spatial resolution of DTI and fMRI used here, this assumption is appropriate and supported by postmortem work investigating human thalamus. The spatial scale of interindividual cytoarchitectonic variation in the spatial extent of thalamic nuclei, described by Morel et al. (1997), is in the order of 2 mm. We account for some anatomical variation and misregistration here by spatially smoothing the individual tractograms. We did this in seed space using a modest 4-mm smoothing kernel, so that the pattern of tractographic whole-brain connectivity from any given thalamic voxel is a weighted average of the patterns from the surrounding thalamic voxels. To keep this minimal smoothing of the thalamus consistent between modalities, the functional time course, corresponding to each resulting independent component, is calculated from the unsmoothed functional data. Cortical functional and architectonic regions are spatially much more variable (Rajkowska and Goldman-Rakic 1995; Uylings et al. 2005) and so the group average functional connectivity with each thalamic region was calculated on data smoothed with a larger 6-mm kernel. In this way, we try and keep to the matched filter theorem, ensuring that we match the spatial smoothness of the data at each step of the analysis to the size of the expected signal and structure. Even with this low level of spatial smoothing, anatomically prominent, but spatially small, structures such as the medial geniculate body may be averaged with the signal from neighboring structures. Their position on the periphery of the thalamus and their relatively small size make them extremely difficult to identify using either fMRI or diffusion tractography without a more specialized approach (e.g., Devlin et al. 2006).

Atlas and morphologically defined regions of interest remain the dominant method of interrogating resting-state connectivity. Though the use of predefined atlases has been useful, population atlases may misrepresent the underlying data. Anatomically defined, they may not reflect functional anatomy, leading to a mixture of incoherent signals being averaged and inadvertently misrepresenting the underlying functional and connectional anatomy (Smith et al. 2011). With this in mind, this study emphasizes that the use of the whole thalamus as a region of interest in connectivity studies is at best troublesome, due to its large functional and structural heterogeneity. Generally, we support the idea of a data-driven parcellation of regions of interest prior to investigations of network properties. The need for this has been shown elsewhere in real (Wang et al. 2009) and simulated data (Smith et al. 2011). The approach shown here is one possible method of utilizing structural connectivity to achieve this. Nonetheless, we only look here at a small region of the brain, and expanding this to cortex and the whole brain remains a computational and methodological challenge.

As illustrated here and elsewhere, resting-state fMRI is an extremely powerful tool to interrogate multiple concurrent functional networks (Biswal et al. 2010). Even though the resolution of fMRI and DTI is coarse relative to the size of individual thalamic nuclei (Morel et al. 1997; Fair et al. 2010), we are here able to demonstrate overlapping patterns of anatomically and functionally meaningful connectivity. Further recruitment by cortical targets during actual cognition is likely to be more extensive through further corticocortical connections. The data provided here reflects connectivity at rest and, though the lack of experimental control is one of the criticisms of rs-fMRI (Kelly et al. 2012), we are here able to delineate functional thalamic networks that shows close correspondence to the connectional anatomy. Although some of the ROIs overlap spatially, their functional correlates are spatially separable, indicating distinct but overlapping thalamic influences on cortical function.

## Conclusion

Using a large population cohort, we have demonstrated the structural and functional connectivity of the thalamus. The results clarify the anatomically constrained functional role of the thalamus in distinct but overlapping cortical networks. Importantly, we illustrate the correspondence of structure to function in a readily interpretable way, revealing a surprisingly small set of anatomically distinct thalamocortical functional modules.

## Supplementary Material

Supplementary material can be found at http://www.cercor.oxfordjournals.org/.

## Funding

J.O.M. is supported by the Wellcome Trust (Grant Number 096195). S.S.K. was supported by a UK Medical Research Council grant (Grant Number MR/K023152/1). M.P.R. is supported by a UK Medical Research Council Programme Grant (Grant Number MR/K013998/1) and the National Institute for Health Research (NIHR) Biomedical Research Centre at the South London and Maudsey NHS Foundation Trust. Funding to pay the Open Access publication charges for this article was provided by the Wellcome Trust.

## Supplementary Material

Supplementary Data
