# Supplementary material for: White Matter Connectivity of the Thalamus Delineates the Functional Architecture of Competing Thalamocortical Systems
Source: Cereb Cortex. 2015 Apr 21;25(11):4477–89. doi: 10.1093/cercor/bhv063 (PMC4816794; doi:10.1093/cercor/bhv063)
Supplement: Supplementary Data [file supp_25_11_4477__index.html]

White Matter Connectivity of the Thalamus Delineates the Functional Architecture of Competing Thalamocortical Systems — Supplementary Data 

# White Matter Connectivity of the Thalamus Delineates the Functional Architecture of Competing Thalamocortical Systems

## Supplementary Data

Supplementary Data

**Files in this Data Supplement:**

- Supplementary Data - Docx file
- Supplementary Table 1 - xlsx file
- Supplementary Table 2 - xlsx file
- Supplementary Table 3 - xlsx file
- Supplementary images1-5 - zip file
