# Supplementary material for: White Matter Connectivity of the Thalamus Delineates the Functional Architecture of Competing Thalamocortical Systems
Source: Cereb Cortex. 2015 Apr 21;25(11):4477–89. doi: 10.1093/cercor/bhv063 (PMC4816794; doi:10.1093/cercor/bhv063)
Supplement: Supplementary Data [file supp_bhv063_bhv063supp.docx]

**Supplementary Table 1**

Dataset IDs, Age and Gender from the NKI-Rockland sample included in this study.

**Supplementary Table 2**

Areas of overlap between functional conjunction maps for each thalamic set and cortical regions as defined by the Harvard-Oxford cortical atlas.

**Supplementary Table 3**

Thalamus parcel order and the set (cluster) to which each parcel is assigned

**Supplementary Image 1**

Mean_Functional.nii.gz : The BOLD-weighted image in MNI space, averaged across subjects.

**Supplementary Image 2**

ICA_Bundles.nii.gz : The raw independent components output from tensor independent component analysis. Images are provided unthresholded. Images are provided in MNI space and sampled at the same resolution of Supplementary Image 1.

**Supplementary Image 3**

ICA_Origin.nii.gz : The Z-normalised weightings of each tractogram that contributed to each independent component, mapped back onto the thalamus. Images are provided unthresholded. Images are provided in MNI space and sampled at the same resolution of Supplementary Image 1.

**Supplementary Image 4**

Functional_Connectivity_Maps.nii.gz : The group average functional connectivity of each of the thalamic seed regions provided in Supplementary Image 3. Each of these images are corrected for multiple comparisons using TFCE and are scaled at 1-p (i.e. a voxel with a corrected p-value of <0.01 would have a value of 0.99 or above in this set of images). Images are provided in MNI space and sampled at the same resolution of Supplementary Image 1.

**Supplementary Image 5**

Functional_Connectivity_Maps_Tstats.nii.gz : The group average functional connectivity of each of the thalamic seed regions provided in Supplementary Image 3. These images are the raw t-score maps and are provided unthresholded. Images are provided in MNI space and sampled at the same resolution of Supplementary Image 1.
